# Correlation between perceived infertility-related stress and fertility quality of life in women diagnosed with primary infertility

**DOI:** 10.1192/j.eurpsy.2021.1602

**Published:** 2021-08-13

**Authors:** B. Batinic, J. Milovanovic, S. Dragojevic-Dikic

**Affiliations:** 1 Clinic Of Psychiatry, Clinical Centre of Serbia, Belgrade, Serbia; 2 Department Of Psychology, Faculty of Philosophy, Belgrade, Serbia; 3 Department Of Obstetrics, Obstetrics and Gynecology Clinic “National front”, Belgrade, Serbia

**Keywords:** infertility-related stress, fertility quality of life, women diagnosed with primary infertility

## Abstract

**Introduction:**

A diagnosis of infertility is a stressful emotional experience for women, leading to a significant detrimental impact in many domains of life quality.

**Objectives:**

The aim of this study is to explore the correlation between perceived infertility-related stress and fertility quality of life in affected women.

**Methods:**

The study sample comprised 236 women diagnosed with primary infertility, recruited from the Gynecology Obstetrics Clinic, with a mean age of 33.21 years (min 20, max 46) and with a mean duration of conception attempts of 3.24 years (min 1, max 16), assessed by the Fertility Problem Inventory (FPI) and the Fertility quality of life questionnaire (FertiQol).

**Results:**

The mean FPI and FertiQol were 137.23 (SD=29.066) and 65.356 (SD=11.119) respectively. There was a significant negative correlation between perceived infertility-related stress and fertility quality of life (r= -.513; p<0.01). All the subscales of the applied questionnaires showed significant negative correlations, with exception of Need for parenthood and Rejection of childfree lifestyle subscales of FPI and Treatment related quality of life of FertiQol. Furthermore, the total FPI score could significantly predict the total FertiQol score (F=83.386; df=1:234; p<0.01). On the basis of perceived infertility-related stress, a 26.3% variance of fertility quality of life can be explained.

**Conclusions:**

Women diagnosed with primary infertility who experience higher levels of infertility-related stress had a lower level of fertility quality of life.

